# Visually induced motion sickness correlates with on-road car sickness while performing a visual task

**DOI:** 10.1007/s00221-025-07020-z

**Published:** 2025-03-03

**Authors:** Tugrul Irmak, Ksander N. de Winkel, Riender Happee

**Affiliations:** 1 Faculty of Mechanical Engineering, Delft University of Technology, Cognitive Robotics, Delft, The Netherlands; 2 Institute for Road Safety Research (SWOV), Den Haag, The Hague, The Netherlands; 3 Nephrology, University Medical Center Utrecht, Utrecht, The Netherlands

**Keywords:** Motion sickness, Visual, Vestibular, VIMS, Individual variations, Susceptibility, Driving, Virtual reality

## Abstract

Previous literature suggests that the motion sickness susceptibility questionnaire (MSSQ) is inadequate for prediction of motion sickness under naturalistic driving conditions. In this study, we investigated whether visually induced motion sickness using a virtual reality headset could be used as a quick and reliable way to predict participant susceptibility. We recruited 22 participants to complete a two-part experiment. In randomised order, we determined their susceptibility to visual motion sickness and their susceptibility to car sickness. To determine visual susceptibility, the visual scene was sequentially rotated at constant velocity around an earth-vertical yaw axis and rolled about the nasiooccipital axis, in 30 s intervals. Car sickness, on the other hand, was elicited under completely naturalistic conditions, being driven in the backseat of a car in the city of Delft, performing a visual task on a laptop. Sickness ratings were collected at regular intervals in both parts of the experiment. We found that the frequencies excited by naturalistic driving are very low, which has important consequences for motion sickness modelling and mitigation in automated vehicles. We found that individual car sickness correlated positively with visual motion sickness. This indicates that both are influenced by a common sickness susceptibility factor. Car sickness correlated similarly with visual motion sickness and MSSQ. Overall, our results indicate that combining measurements of sickness responses to a visual stimulus and MSSQ can yield a reliable method for determining individual sickness susceptibility. To this end the visual stimulus and the weighting with MSSQ responses can be refined using a much larger sample and considering additional visual conditions in driving.

## Introduction

Automated vehicles are presented as a revolutionary new technology that will shift how the average vehicle is utilised. This shift will be in the form of a turn away from “driving” to “riding”. The aim is that the former driver, that is now a passenger, is freed from the driving task and can use the travel time for other activities. A major problem, however, is that whereas drivers rarely get motion sick, 46% of passengers do (Schmidt et al. 2020); particularly in eyes-off-road conditions (Irmak et al. 2021a).

As motion sickness severely limits the ability to engage in other activities, this reduces the utility of vehicle automation. Several current research efforts aim to develop mitigation methods that can be used to reduce the incidence of motion sickness (Winkel et al. 2021; Karjanto et al. 2018; Kuiper et al. 2020). Such mitigation methods include’comfortable driving styles’, through smart strategies like motion planning, path planning, route planning and active suspension (Zheng et al. 2024, 2023; Jain et al. 2023) to minimize the provocative stimulus. Other mitigation methods augment perception rather than augmenting the actual vehicle motions. These include interfaces increasing anticipation of vehicle motions via auditory (Kuiper et al. 2020), visual (Karjanto et al. 2018; Winkel et al. 2021) and tactile (Li and Chen 2022) cues; or decreasing reliance on the vestibular senses by noisy vibrations. Assessments of the effectiveness of such mitigation methods requires experiments where human participants are subjected to provocative motions. The efficacy of the mitigation method under study can then be determined by contrasting the level of sickness attained in an experimental condition with the mitigation method to a control condition without.

Detrimental to the generalizability of such studies over a target population is the extent of noise in sickness measurements, which is primarily due to interpersonal variability in overall motion sickness susceptibility (Irmak et al. 2021b). This means that some participants may not respond to the sickening stimulus at all, whereas others may respond too fast. In such cases, there is a flooring or a ceiling response, which limits an experiment’s ability to differentiate between conditions.

In order to make efficient use of time and resources, it is useful to select a representative sample of participants. The conventional method of participant selection is through the Motion Sickness Susceptibility Questionnaire (MSSQ) (Golding 2006). The MSSQ queries a participant’s historical experience of motion sickness on various modes of transport. Here, the sum of the scores yields a (self reported) representation of individual susceptibility. Many studies present MSSQ to control and characterize the population tested but do not report correlations to sickness. Unfortunately, some studies have indicated MSSQ to be an unreliable predictor of individual susceptibility to on-road car sickness. For simple laboratory experiments a correlation of 0.45 was found averaged over ten studies (Golding 1998). However, for passive driving (Muhlbacher et al. 2020) reports a Spearman rank correlation of only 0.212 between MSSQ Total and MISC and 0.266 between MSSQ Adult and MISC. In our slalom experiment with passive driving (Irmak et al. 2021a) the correlation was 0.27 (*p* = 0*.*29) eyes-off road and 0.54 (*p* = 0*.*02) eyes-on road (this correlation derives from the original data and was not in the published paper (Irmak et al. 2021a)). A low correlation was also found in a ship simulator (Bos et al. 2005).

Reasons for the limited predictability of MSSQ may be that the MSSQ pools reports of susceptibility to multiple stimulation modes, and the limited reliability of self reports, due to various cognitive and memory biases (Paulhus 1991).

Another way to gauge susceptibility to car sickness may be to expose participants to a short duration of sickening motions. In experiments by Irmak et al. (2022) it was observed that fore-aft sinusoidal accelerations at 0.3 Hz and 2.5 ms^−2^ for on average 8 min correlated strongly with overall motion sickness susceptibility to fore-aft motions of much longer duration. However, as these experiments were performed using a simulator with a large motion base (SIMONA flight simulator motion platform, TU Delft) this specific method is not cost- and time-efficient, prohibiting wide scale adoption. Instead, a more accessible pretest for assessing sickness susceptibility may be using a provocative visual stimulus.

To the best of our knowledge, there is no study that directly investigates the relationship between visually induced motion sickness and car sickness.

A relevant and promising study (Mazloumi Gavgani et al. 2018) compares a simple motion paradigm on a rotational seat (cross-coupled coriolis) with eyes closed, to a vision only condition being a virtual reality ride on a roller coaster. In this study the individual maximum nausea rating in the two conditions was highly correlated (r = 0.58, *p* < 0*.*001).

In Bijveld et al. (2008) off-vertical axis rotation (OVAR) with and without vision was compared to a corresponding vision only condition. This paper does not report whether individual susceptibility correlates across motion and/or visual stimulus conditions. However, it presents an interesting relation with the individual MSSQ, where subjects with low MSSQ, developed nausea more slowly with vision (comparing OVAR with vision to OVAR without vision, *r* = 0*.*7*, p* < 0*.*05). They hypothesize that*”subjects who fared better in the light used visual cues to resolve sensory conflict, whereas subjects who were equally susceptible in light and dark made poor use of visual cues. This may explain why some people prefer ‘a view of the road ahead’ to help against motion sickness whereas others shut their eyes”*.

Studies have shown that genetic factors explain half the variability in sickness susceptibility (Reavley et al. 2006). Moreover, there is a relationship between higher motion sickness susceptibility and greater susceptibility to chemotherapy induced nausea (Golding 1998), and similar observations have been made for other sources of non-motion based emetic stimuli; even in animal studies (Golding 2006). Overall, these findings indicate the existence of a general sickness susceptibility factor, in addition to other specific factors, i.e., habituation/prior motion experience. Therefore, based on the expectation of a general sickness susceptibility factor, susceptibility to visually induced motion sickness is likely to be correlated with car sickness encountered in real traffic. Indeed, survey results have shown MSSQ scores to correlate positively with the visually induced motion sickness susceptibility questionnaire (VIMSSQ) (Golding et al. 2021).

In the present study, we will experimentally assess the correlation between susceptibility to visually induced motion sickness and motion sickness induced in a naturalistic drive. We will evaluate the usefulness of the visual stimulus as predictor of susceptibility to car sickness for future motion sickness experiments that may supplant, or be used in tandem with, the currently used MSSQ. We will do this by having participants perform a two-part experiment. In the first part, we will determine the susceptibility to visually induced motion sickness. The visual stimuli will be in the form of pseudo-Coriolis stimulation provided using a virtual reality headset. This stimulation is very intense, and is expected to provoke quick motion sickness responses (Dichgans and Brandt 1973; Bonato et al. 2009). In the second experiment, susceptibility to car sickness will be determined. This will be done by driving participants along rural and urban roads around the city of Delft in conditions that mimic a typical commute. Based on the literature, we hypothesize that:

1. Visually induced motion sickness correlates positively with car sickness.
2. Susceptibility to visual motion stimuli is a better participant selection criterion than the motion sickness susceptibility questionnaire.

## Methods

### A. Participants

In total, 22 participants completed this study (mean age: 26.1 years, STD: 10.8, 10 female, 12 male). Before the experiment participants were asked to fill in the MSSQ (short) (Golding 2006). The 22 participants had a median motion sickness susceptibility MSSQ-Short score of 11.3, corresponding to the 50*th* percentile. This indicates that they were of average motion sickness susceptibility.

### B. Apparatus & stimulus

**Fig. 1:**
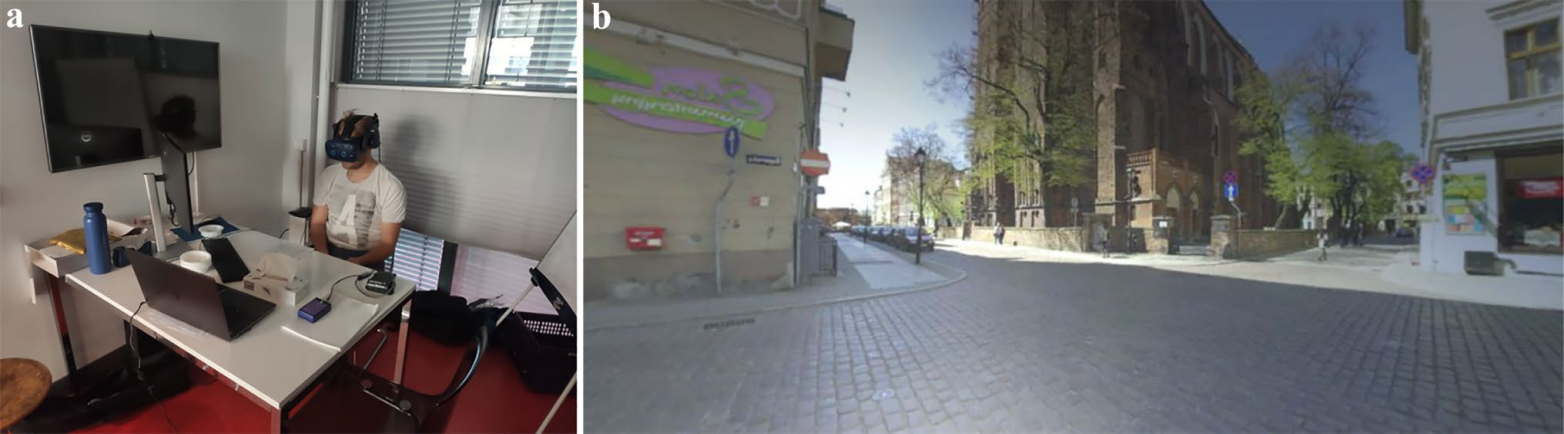
Setup of the virtual reality experiment. **a** shows the participant seated with an erect posture, on a static chair with the headset attached, viewing the visual stimulus. There were two electrodes attached to the fingers which recorded GSR. **b** shows the virtual world from the point of view of the participant

*1) Virtual Reality Experiment:* This experiment was performed using an HTC Vive virtual reality headset. During the experiment, participants wore the headset and were seated on a chair in a quiet room (see Fig. 1a). Participants kept an erect posture while looking ahead. The scene presented to the participant in the virtual reality environment consisted of a natural urban scene (see Fig. 1). The visual world was rotated around a yaw-axis at a constant angular velocity of 150^◦^/s. Every 30 s this axis was tilted away from the earth vertical by 40^◦^. This angle was held for 20 s and then rotated back to the vertical (see Fig. 1). There were two electrodes attached to the fingers which recorded the Galvanic Skin Reaction (GSR) using a Nexus 4 with the Biotrace NX4 software. Participants were instructed to report their sickness on the 11-point MISC scale (Bos et al. 2010; Winkel et al. 2022), which was shown and explained before the experiment. The MISC scale is anchored to specific motion sickness symptoms: 0 is no symptoms, 1 is uneasiness, 2, 3, 4, 5 represent increasing severity of non-nausea symptoms from vague to severe, 6 is mild nausea, 7 is moderate nausea, 8 is severe nausea with 9 and 10 being retching and vomiting, respectively. There was a 1 kHz beep every 20 s which prompted the participants to give their MISC scores. Responses were recorded on audio and transcribed after the experiment session by the experimenter. The audio recordings were voice activated and recorded only for the duration the participant was speaking. Each MISC rating given by the participant was time stamped to the start of the audio sample. The visual stimulus was presented for a maximum duration of 10 min, or until the participants reached a MISC of 6.

**Fig. 2:**
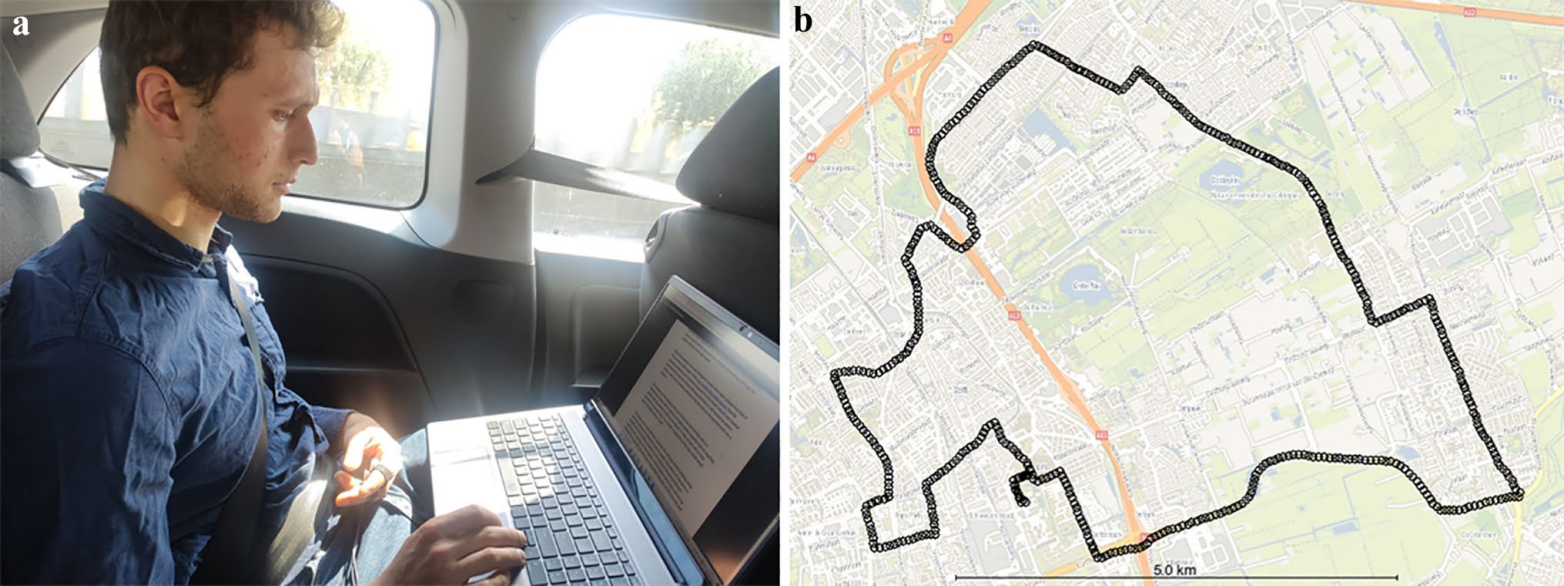
Setup of the car experiment. **a** shows the participant (first author) seated on the back seat of the car, with a laptop on his lap, reading the given texts. There were two electrodes attached to the fingers which recorded GSR. **b** shows the GPS track superimposed on to the map of Delft. This was a naturalistic drive through rural and urban areas

*2) Car Experiment:* In this part of the experiment, the participants were seated in the right back seat of a five-seat passenger car. They had a laptop on their lap, from which they read short texts that were given in randomised order to each participant. The participants were instructed to read the texts in front of them. Therefore, the visual condition can be described as internal vision, with some peripheral visual cues. As with the vision experiment, GSR was recorded with the electrodes attached to the index and middle fingers of the left hand. Participants wore a seat belt at all times. The participants were driven on urban and rural roads in and around the city of Delft, the Netherlands (see Fig. 2b). All tests were performed by the same driver. The final route was chosen subjectively among three candidate drives. The vehicle was equipped with an IMU (Xsens Movella DOT) attached to the floor of the vehicle recording 3D translational accelerations at 100 Hz. Accelerations were transformed to the frequency domain using Welch’s method. GPS position was recorded with a medium accuracy system, but not used in this paper. There was a 1 kHz beep every 30 s which prompted the participants to give their sickness scores on the MIsery Scale (MISC). The MISC scale was shown and explained before the drive and was visible during the experiment being placed on the seat left of the participants. The drive continued until the participants finished the whole route, or until they twice reached a MISC of 6. In the first instance a MISC of 6 was reached, the vehicle was safely pulled aside and a minimum of 5 min was given for the participant to recover to a MISC of 2. The drive was then resumed. If the participant reached a MISC score of 6 a second time, the vehicle was again stopped, recovery was allowed and the vehicle was driven back to the staging ground as carefully as possible as to elicit very little sickness.

After the VR and the car experiment participants filled in the motion sickness assessment questionnaire (MSAQ) (Gianaros et al. 2001).

For each participant and each condition we evaluated, (1) the end time *t*_*end*_, being the first time of reaching MISC 6 or the actual end time when this level was not reached, (2) the”mean MISC” over time up to *t*_*end*_, (3) the”maximum MISC”.

## Results

In this section, the sickness results, the vehicle accelerations and the correlations between visual and car sickness are presented.

### A. Observed sickness

**Fig. 3:**
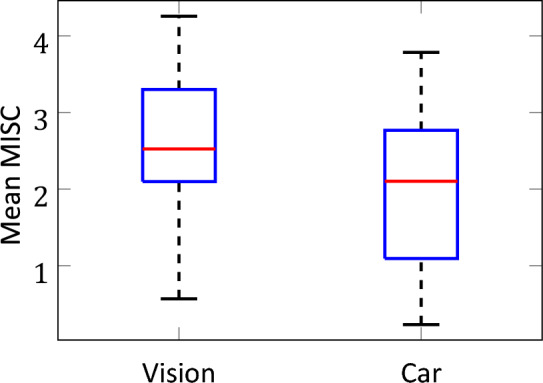
Mean MISC over time in the vision experiment compared to the car experiment. The red lines indicate the medians. Wilcoxon signed rank tests support a significant difference in sickness between the two conditions

In this study, the MISC indicated a greater level of sickness for the vision condition (median mean MISC = 2.52) compared to the naturalistic car condition (median mean MISC = 2.10) (Fig. 3). This is despite the much shorter exposure duration of 10 min, compared to the much longer exposure duration in the car of 60 min. The higher sickness in the vision condition was significant for mean MISC (*p* = 0*.*044), maximum MISC (*p* = 0*.*035) and MSAQ (*p* = 0*.*018) according to a Wilcoxon signed-rank test.

### B. Vehicle motions

**Fig. 4:**
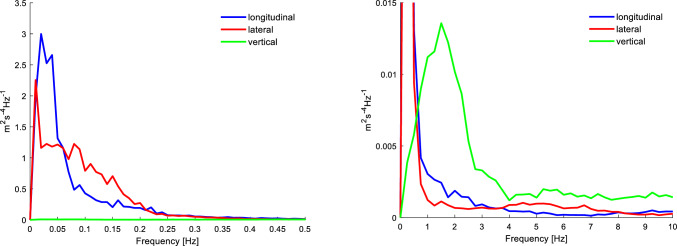
Mean Welch power spectral density estimates for 16 participants (for whom the IMU data were available) showing spectral content of longitudinal (blue), lateral (red), and vertical (green) vehicle acceleration, up to 0.5 Hz (left) and up to 10 Hz (right)

The motions encountered by the participant contribute to their subsequent ratings. Here, prime contributors were the longitudinal and the lateral accelerations of the vehicle. As shown in Fig. 4, the longitudinal accelerations peak sharply between 0.01 and 0.04 Hz. The lateral accelerations, on the other hand, are wider band, with a plateau between 0.01 and 0.1 Hz. Despite the naturalistic drive having a large urban component, with the associated speed bumps, the vertical accelerations were minor in particular at the lower frequencies associated with motion sickness. The fact that our sensitivity to vertical motion is similar to horizontal motion (Donohew and Griffin 2004; Bos et al. 2024) indicates that vertical accelerations encountered during naturalistic development of car sickness on relatively flat roads as studied in this experiment do not meaningfully contribute to its increase.

### C. Linear regression model

Table 1 shows correlations between sickness in car and VR experiments and self reported susceptibility (MSSQ). Here the inverse termination time 1*/t*_*end*_ is presented instead of the actual *t*_*end*_. The inverse is seen as more intuitive and is positively related to sickness susceptibility. Using the inverse improves correlations where 1*/t*_*end*_ has a correlation of 0.81 between car and VR while *t*_*end*_ sees a correlation of 0.51.

**Table 1:** Correlations between sickness in car and VR experiments (MISC, 1*/t*_*end*_ and MSAQ) and self reported susceptibility (MSSQ); *t*_*end*_ is the time of reaching MISC = 6 or ending the experiment with lower MISC levels at the scheduled time

	mean MISC car	max MISC car	1*/t*_*end*_ car	MSAQ car	mean MISC VR	max MISC VR	1*/t*_*end*_ VR	MSAQ VR	MSSQ
mean MISC car	1.00	0.88	0.14	0.65	0.34	0.27	0.10	0.25	0.45
max MISC car	0.88	1.00	0.43	0.65	0.35	0.44	0.40	0.36	0.54
1*/t*_*end*_ car	0.14	0.43	1.00	0.47	-0.05	0.20	0.81	0.38	0.30
MSAQ car	0.65	0.65	0.47	1.00	0.13	0.14	0.32	0.51	0.60
mean MISC VR	0.34	0.35	-0.05	0.13	1.00	0.79	0.17	0.04	0.29
max MISC VR	0.27	0.44	0.20	0.14	0.79	1.00	0.51	0.18	0.37
1*/t*_*end*_ VR	0.10	0.40	0.81	0.32	0.17	0.51	1.00	0.37	0.45
MSAQ VR	0.25	0.36	0.38	0.51	0.04	0.18	0.37	1.00	0.38
MSSQ	0.45	0.54	0.30	0.60	0.29	0.37	0.45	0.38	1.00

All relevant correlations are positive indicating positive relations between sickness in car and VR and MSSQ. The MSSQ correlates reasonably well to sickness for these experiments with somewhat higher correlations for car as compared to VR conditions. The car results also correlate reasonably well to the VR results with the highest correlation (0.81) observed for the inverse end time.

To assess how predictive the MSSQ and the susceptibility to the visual stimulus was of susceptibility to car sickness, a linear regression model was fitted to the aggregate group data. This was done using the MATLAB function *fitlm*. A model of the form *MSAQ*_*car*_ = *a MSAQ*_*vis*_ + *b* was used to evaluate the hypothesis that*”visually induced motion sickness relates positively with car sickness”*. Here, the coefficient *a* = 0*.*40 is significantly different from zero (*p* = 0*.*016) with *R*^2^ = 0*.*26. The corresponding Pearson’s correlation coefficient was *ρ* = 0*.*51 (*p* = 0*.*016). Therefore, there is sufficient evidence to conclude that individual susceptibility to visually induced motion sickness does correlate positively with car sickness. However, it is seen from a model of the form *MSAQ*_*car*_ = *a MSSQ* + *b* that MSSQ is also predictive of susceptibility to car-sickness with the coefficient *a* = 0*.*781 (*p* = 0*.*0034) with an *R*^2^ = 0*.*35 and a corresponding Pearson’s correlation coefficient of *ρ* = 0*.*595.

A joint linear model of the form *MSAQ*_*car*_ = *a MSAQ*_*vis*_ + *b MSSQ* + *c* has better fit performance (*R*^2^ = 0*.*45) than any single one of the individual predictors on their own. In this case, the coefficients were *a* = 0*.*26, *b* = 0*.*62 and *c* = 10*.*761. However, in this model, the contribution of the *MSAQ*_*vis*_ was not significant (*p* = 0*.*091). Similar models were fitted for mean MISC, max MISC and 1*/t*_*end*_ in the car experiment. For mean MISC and max MISC only MSSQ was significant (*p* = 0*.*04 and 0*.*009 respectively). For 1*/t*_*end*_ in the car only 1*/t*_*end*_ in VR was predictive (*p* < 0*.*001). These were indeed the only significant effects as verified by stepwise regression using all VR results and MSSQ to predict sickness in the car experiment. Therefore, for the VR stimulus now used, we find no clear support for the second hypothesis:*”susceptibility to visual motion stimuli is a better participant selection criterion than the motion sickness susceptibility questionnaire”*.

**Fig. 5:**
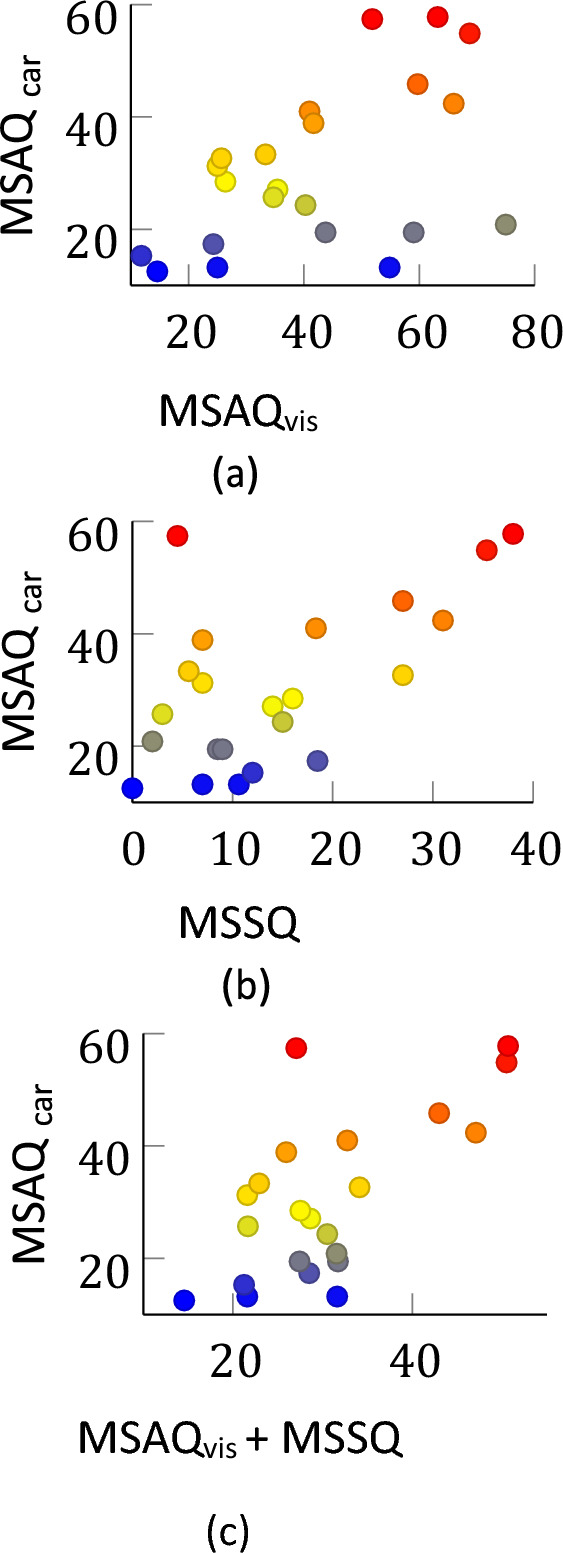
MSAQ in the car as function of MSAQ in the visual experiment **a**, as function of MSSQ **b** and a joint metric using both visual MSAQ and MSSQ **c**, where color scales with MSAQ_car_

Figure 5 shows the scatter plots of sickness encountered in the car, denoted by *MSAQ*_*car*_ against that encountered in the visual stimulus *MSAQ*_*vis*_, selfreported susceptibility *MSSQ* and a linear summation of *MSAQ*_*vis*_ and *MSSQ*. Qualitative evaluation of the plots shows that for the visual stimulus (Fig. 5a) three participants were much more sensitive to the visual stimulus than to the inertial stimulus. Interestingly, all participants that were very susceptible to car sickness were also very susceptible to visually induced motion sickness (Fig. 5a).

## Discussion

For the first time, this study investigated the correlation between visually induced motion sickness and car sickness. Participants underwent a two-part experiment where their susceptibilities to both visually induced motion sickness, and to car sickness under naturalistic conditions were measured. We then evaluated whether the visual stimulus experiment could supplant the MSSQ questionnaire as a preselection tool. In the following, we discuss our findings in relation to the hypotheses and consider the statistics of naturalistic driving and its potential influence on sickness susceptibility.

### A. Vehicle accelerations

In this study, we found that vehicle accelerations encountered during naturalistic manual driving were generally narrow bandwidth and low frequency. Longitudinal accelerations peak sharply between 0.01 and 0.04 Hz. The lateral accelerations peak between 0.01 and 0.1 Hz. Experiments on a suburban route (Griffin and Newman 2004) show similar frequency distributions with substantial differences in amplitude, rather than frequency between vehicle types and between drivers.

This makes modelling car sickness, at least for trajectory control for automated vehicles, easier and more computationally efficient, reducing the need for complex motion sickness models. Such motion sickness models would then only need to be accurate in the frequency ranges of interest in driving. This could combine simple filters like the motion sickness dosage value (MSDV) with an Oman model of nausea to predict the time evolution of sickness (Irmak et al. 2022) and could include individual susceptibility variations (Kotian et al. 2023; Kotian et al. in press; Metzulat et al. 2024).

Prior experiments (Irmak et al. 2021b) indicated that individuals have their own specific frequency sensitivity curves. Exploring individual frequency sensitivity across a representative population requires major experimental efforts. A recent study (Bos et al. 2024) tested 0.06, 0.1, 0.2, 0.4, 0.8, 1.6 and 3.2 Hz in fore-aft, 0.06, 0.1, 0.2, 0.4, and 0.8 Hz in lateral, and 0.24, 0.2, 0.4, 0.8, 1.6 and 3.2 Hz in vertical motion. To assess individual susceptibility to car sickness we would apparently need to focus on the lower frequencies.

### B. Predicting car sickness from VR and MSSQ

In this study, we found that the vision paradigm we used was, on average, more sickening than car sickness (mean MISC 2.52 vs 2.10). But because the vision paradigm occurred over an exposure duration of 10 min, compared to the 60 min of the car, it likely elicits more sensory conflict. In terms of correlations, we see that visually-induced motion sickness susceptibility correlates moderately with car sickness magnitude (*ρ* = 0*.*51) for MSAQ and correlates well (*ρ* = 0*.*81) for 1*/t*_*end*_). Because the two perturbations are very distinct, such a correlation supports the notion of a general sickness susceptibility factor. This susceptibility factor is likely an idiosyncratic property. However the visual susceptibility on its own is not yet discriminative enough to perform better than MSSQ as a participant selection tool. We see from Fig. 5 that no one who scored low on the visual motion sickness, scored high on the car sickness, likewise everyone who scored high on the MSSQ scored high on car sickness. This indicates that the visual and MSSQ are discriminative at different points of the susceptibility curve. Therefore, a combined metric using MSSQ and visually-induced sickness susceptibility (Fig. 5c) likely provides a more robust correlate of on-road susceptibility.

In future studies, the visual stimulus can be adapted in terms of yaw rotational velocity, tilt angle, timing, and predictability. Experiments could vary such parameters to achieve a better correlation with car sickness. Stimulus timing could be adapted to better reflect the bandwidth of longitudinal and lateral vehicle accelerations. The visual stimulus used in our experiment had a periodicity of 130 s corresponding to a frequency of 0.008 Hz. This may be increased to better match the dominant frequency in the car experiment being 0.01–0.04 Hz for longitudinal and 0.01–0.1 Hz for lateral motion. The end time of the visual test correlated most strongly with the car test. However most participants did not reach the termination criterion (MISC = 6). Duration and amplitude of the visual test can be increased, making the end-time an even more informative measure.

Lastly, an advantage of the visual stimulus over the MSSQ is that individual specific Oman model time constants can be identified, which allows for not only a pre-test measurement of susceptibility, but also the temporal dynamics of sickness, including hypersensitivity and rest. The disadvantage of course is that it would take ten minutes of excitation some days prior to the actual experiment, and so has a smaller throughput than the MSSQ.

### C. Limitations

This paper evaluated individual susceptibility to car sickness while reading on a laptop. Future studies shall quantify how results translate to other visual and motor task conditions. A recent paper (Metzulat et al. 2024) cites relevant papers on the effect of NDRTs and report somewhat increased sickness with a visual dynamic task compared to an auditory task.

The MSSQ has been extensively validated and refined using large and representative populations. Likewise our visual stimulus and the proposed integration with MSSQ can be validated and refined. Here a larger and more representative sample will allow non-linear weighting towards a well validated and robust car sickness prediction.

## Conclusions

This study investigated the correlation between visually induced motion sickness and car sickness. We found that the accelerations encountered during naturalistic driving were generally very low frequency, simplifying sickness modelling for vehicular motions. Moreover, we found that visually-induced motion sickness correlated positively with car sickness. This supports the existence of an individual specific general sickness-susceptibility factor. Lastly, the visual stimulus was not a better predictor of motion sickness susceptibility in the car than the MSSQ. However, using both jointly as predictors with a better optimised visual stimulus may provide a robust and highly selective participant preselection tool supporting effective testing in research and development mitigating self-driving car sickness.

## Data Availability

Experimental data are supplied in: https://doi.org/10.4121/5f54188f-9e47-4ac7-8cf3-2ebb852bdf15

## References

[CR1] Bijveld M, Bronstein AM, Golding JF, Gresty MA (2008) Nauseogenicity of off-vertical axis rotation vs. equivalent visual motion. Aviat Space Environ Med 79(7):661–665 18619124 10.3357/asem.2241.2008

[CR2] Bonato F, Bubka A, Palmisano S (2009) Combined pitch and roll and cybersickness in a virtual environment. Aviat Space Environ Med 80(11):941–945 19911517 10.3357/asem.2394.2009

[CR3] Bos JE, MacKinnon SN, Patterson A (2005) Motion sickness symptoms in a ship motion simulator: effects of inside, outside, and no view. Aviat Space Environ Med 76(12):1111–1118 16370260

[CR4] Bos JE, de Vries SC, van Emmerik ML, Groen EL (2010) The effect of internal and external fields of view on visually induced motion sickness. Appl Ergon 41(4):516–521 20042182 10.1016/j.apergo.2009.11.007

[CR5] Bos JE, Souman JL, Nooij S, Diels C (2024) Advancing ISO 2631–1 by considering pre-emesis symptoms in carsickness. In *Proceedings of the Driving Simulation Conference*, Strassbourg, France [Online]. Available: https://proceedings.driving-simulation.org/proceeding/dsc-2024/advancing-iso-2631–1-by-considering-pre-emesis-symptoms-in-carsickness

[CR6] de Winkel KN, Pretto P, Nooij SA, Cohen I, Bulthoff HH (2021) Efficacy of augmented visual environments for¨ reducing sickness in autonomous vehicles. Appl Ergon 90:103282 33065467 10.1016/j.apergo.2020.103282

[CR7] de Winkel KN, Irmak T, Kotian V, Pool DM, Happee R (2022) Relating individual motion sickness levels to subjective discomfort ratings. Exp Brain Res 240(4):1231–1240 35192043 10.1007/s00221-022-06334-6 PMC8861616

[CR8] Dichgans J, Brandt T (1973) Optokinetic motion sickness and pseudo-coriolis effects induced by moving visual stimuli. Acta Otolaryngol 76(1–6):339–348 4543918 10.3109/00016487309121519

[CR9] Donohew BE, Griffin MJ (2004) Motion sickness: effect of the frequency of lateral oscillation. Aviat Space Environ Med 75(8):649–656 15328780

[CR10] Gianaros PJ, Muth ER, Mordkoff JT, Levine ME, Stern RM (2001) A questionnaire for the assessment of the multiple dimensions of motion sickness. Aviat Space Environ Med 72(2):115 11211039 PMC2910410

[CR11] Golding JF (1998) Motion sickness susceptibility questionnaire revised and its relationship to other forms of sickness. Brain Res Bull 47(5):507–516 10052582 10.1016/s0361-9230(98)00091-4

[CR12] Golding JF (2006) Motion sickness susceptibility. Auton Neurosci 129(1–2):67–76 16931173 10.1016/j.autneu.2006.07.019

[CR13] Golding JF, Rafiq A, Keshavarz B (2021) Predicting individual susceptibility to visually induced motion sickness by questionnaire. Front Virtual Reality 2:576871

[CR14] Griffin M, Newman M (2004) An experimental study of low-frequency motion in cars. Proceed Instit Mech Eng, Part D: J Automob Eng 218(11):1231–1238

[CR15] Irmak T, Pool DM, Happee R (2021a) Objective and subjective responses to motion sickness: the group and the individual. Exp Brain Res 239:515–531 33249541 10.1007/s00221-020-05986-6 PMC7936971

[CR16] Irmak T, de Winkel KN, Pool DM, Bulthoff HH, Happee R (2021b) Individual motion perception parameters and motion sickness frequency sensitivity in fore-aft motion. Exp Brain Res 239(6):1727–1745 33779793 10.1007/s00221-021-06093-w PMC8006642

[CR17] Irmak T, Kotian V, Happee R, de Winkel KN, Pool DM (2022) Amplitude and temporal dynamics of motion sickness. Front Syst Neurosci. 10.3389/fnsys.2022.866503 35615427 10.3389/fnsys.2022.866503 PMC9126086

[CR18] Jain V, Kumar S, Papaioannou G, Happee R, Shyrokau B (2023) Optimal trajectory planning for mitigated motion sickness: Simulator study assessment. IEEE Transact Intell Transport Syst. 10.1109/TITS.2023.3281724

[CR19] Karjanto J, Yusof NM, Wang C, Terken J, Delbressine F, Rauterberg M (2018) The effect of peripheral visual feedforward system in enhancing situation awareness and mitigating motion sickness in fully automated driving. Transport Res Part F: Traffic Psychol Behav 58:678–692

[CR20] Kotian V, Pool DM, Happee R (2023) Modelling individual motion sickness accumulation in vehicles and driving simulators,” In: *Proceedings of the Driving Simulation Conference*. Antibes, France

[CR21] Kotian V, Pool DM, Happee R, Personalising motion sickness models: estimation and statistical modeling of individual-specific parameters. Provisionally accepted. *Front Neurosci*. 10.3389/fnsys.2025.1531795 10.3389/fnsys.2025.1531795 PMC12206751 40589464

[CR22] Kuiper OX, Bos JE, Diels C, Schmidt EA (2020) Knowing what’s coming: Anticipatory audio cues can mitigate motion sickness. Appl Ergon 85:103068 32174356 10.1016/j.apergo.2020.103068

[CR23] Li D, Chen L (2022) Mitigating motion sickness in automated vehicles with vibration cue system. Ergonomics 65(10):1313–1325 35020579 10.1080/00140139.2022.2028902

[CR24] Mazloumi Gavgani A, Walker FR, Hodgson DM, Nalivaiko E (2018) A comparative study of cybersickness during exposure to virtual reality and “classic” motion sickness: are they different? J Appl Physiol 125:1670–1680 30284516 10.1152/japplphysiol.00338.2018

[CR25] Metzulat M, Metz B, Landau A, Neukum A, Kunde W (2024) Does the visual input matter? Influence of non-driving related tasks on car sickness in an open road setting. Transport Res Part F: Traffic Psychol Behav 104:234–248

[CR26] Muhlbacher D, Tomzig M, Reinmuller K, Rittger L (2020) Methodological considerations concerning motion sickness investigations during automated driving. Informat. 10.3390/info11050265

[CR27] Paulhus DL (1991) Measurement and control of response bias. Elsevier, NY

[CR28] Reavley CM, Golding JF, Cherkas LF, Spector TD, MacGregor AJ (2006) Genetic influences on motion sickness susceptibility in adult women: a classical twin study. Aviat Space Environ Med 77(11):1148–1152 17086768

[CR29] Schmidt EA, Kuiper OX, Wolter S, Diels C, Bos JE (2020) An international survey on the incidence and modulating factors of carsickness. Transport Res Part F: Traffic Psychol Behav 71:76–87

[CR30] Zheng Y, Shyrokau B, Keviczky T (2024) Mitigating motion sickness with optimization-based motion planning. IEEE Transact Intell Vehicles 9(1):2553–2563

[CR31] Zheng Y, Shyrokau B, Keviczky T (2023) Comfort-oriented driving: performance comparison between human drivers and motion planners. [Online]. Available: https://arxiv.org/abs/2301.10538

